# Common Core Bacterial Biomarkers of Bladder Cancer Based on Multiple Datasets

**DOI:** 10.1155/2019/4824909

**Published:** 2019-06-19

**Authors:** Guoqin Mai, Limei Chen, Ran Li, Quan Liu, Haoran Zhang, Yingfei Ma

**Affiliations:** ^1^Institute of Synthetic Biology, Shenzhen Institutes of Advanced Technology, Chinese Academy of Sciences, Shenzhen, China; ^2^Shenzhen Institute of Synthetic Biology, Shenzhen Institutes of Advanced Technology, Chinese Academy of Sciences, Shenzhen, China; ^3^College of Chemistry and Environmental Engineering, Shenzhen University, Shenzhen, Guangdong 518060, China

## Abstract

Recent studies have shown that microorganisms may be associated with the onset and development of bladder cancer. The purpose of this study is to identify the common core bacteria associated with bladder cancer. We characterized the urinary microbial profile of the individuals with bladder cancer by 16S rRNA gene sequencing, and the results of 24 bladder cancer samples collected in our laboratory reveal 31 common core bacteria at genera level. In addition, the abundance of four common core bacteria is significantly higher in bladder cancer samples than in samples from nondiseased people analyzed by LEfSe, based on two previous datasets. In particular, the abundance of* Acinetobacter* is much higher in bladder cancer samples. It has been reported that* Acinetobacter *is involved not only in biofilm formation but also in the adhesion and invasion of epithelial cells, the spread of bacteria caused by the degradation of phospholipids in the mucosal barrier, and the escape of the host immune response. Thus,* Acinetobacter* may be related to bladder cancer and is a potential microbial marker of bladder cancer. However, due to the limited number of participants, further studies are needed to better understand the role of microorganisms in bladder cancer to provide novel biomarkers for diagnosis, prognosis, and therapy.

## 1. Introduction

Bladder cancer is the ninth most common malignant tumor. Every year, more than 430,000 patients are diagnosed with bladder cancer, and over 160,000 people die of bladder cancer globally [1, 2]. In the past few decades, bladder cancer has attracted the attention of scientists for its high incidence and mortality rate. However, the etiology and pathophysiology of bladder cancer remain unclear. It may be caused by genetic mutations and external risk factors, including smoking, exposure to carcinogens, chlorination of drinking water, and cyclophosphamide treatment [3]. In addition to environmental and genetic factors, researchers are increasingly aware that microorganisms in the human body play an important role in maintaining health and developing disease. Microorganisms affect the physiological functions of the human body, such as metabolism, immunity, and hematopoiesis [4]. Transient inflammation is considered part of the body's immune defense against pathogens, but persistent inflammation may promote the development of cancer [5]. Epidemiological studies have shown that chronic inflammation makes people susceptible to various cancers [6]. It is estimated that infections and inflammatory responses are associated with 15% to 20% of all deaths from cancer globally [7]. Studies suggest that microbial dysbiosis may promote the development of some malignant tumors, such as colorectal cancer and breast cancer [8, 9]. Even the links between certain pathogens and cancer have been well established, for example,* Helicobacter pylori* and gastric cancer [10]. In recent years, urinary microflora in urothelial bladder cancer has been studied. Xu et al. reported that* Streptococcus* spp. are more abundant in urine from bladder cancer patients (*n* = 8) compared to healthy individuals (*n* = 6) [11]. Bucevic et al. found enrichment of some bacteria (e.g., the genera* Fusobacterium*,* Actinobaculum*,* Facklamia*,* Campylobacter*, and* Subdoligranulum* and the family* Ruminococcaceae*) in urine from bladder cancer patients (*n* = 12) compared to healthy individuals (*n* = 11) [1]. Wu et al. found enrichment of some bacterial genera (e.g.,* Acinetobacter*,* Anaerococcus*, and* Sphingobacterium*) in urine from bladder cancer patients (*n* = 31) compared to nonneoplastic controls (*n* = 18) [2]. However, the biomarkers found in the bladder cancer samples of the above three laboratories are different. Therefore, in this study we investigate the common core bacteria in 24 bladder cancer samples collected in our laboratory. We have found that the abundance of some common core bacteria is significantly higher in bladder cancer samples than in samples from nondiseased people (based on two previous datasets [1, 2]), providing insight into the role of the microbiome in bladder cancer.

## 2. Results and Discussion

### 2.1. Bladder Cancer Patients and Sequencing Data

Urine samples were collected from 25 patients with bladder cancer. One sample failed to meet the quality standard because of its low sequencing depth. The characteristics of 24 patients with bladder cancer analyzed in this study are presented in Table S1. Eighteen male patients and six female patients were included, ranging from 30 to 86 years. High-throughput sequencing of urine samples from patients with bladder cancer yielded a total of 2,604,140 raw sequences, which were then merged into 1,200,568 paired sequences. After quality filtering and label classification, the number of sequences was reduced to 368,525, with an average read length of 283 base pairs. They were assigned to 4100 operational taxonomic units (OTUs) at 97% similarity by QIIME [12]. Rarefaction curves showed that the 24 samples were sequenced to a sufficient depth, and most samples have captured complete microbial profiles (Figure S1). A total of 26 phyla, 60 classes, 114 orders, 217 families, and 422 bacterial genera were identified, averagely 132.4 genera per sample. A total of 26 phyla, 60 classes, 114 orders, 217 families, and 422 bacterial genera were identified, averagely 132.4 genera per sample. Raw data of bladder cancer and healthy control samples from other laboratories [2, 13] were also analyzed by using QIIME [12]. In the raw data by Wu et al. [2], a total of 34 phyla, 93 classes, 158 orders, 297 families, and 646 bacterial genera were identified, averagely 90 genera per sample. In the raw data by Bucevic et al. [1], a total of 19 phyla, 35 classes, 59 orders, 147 families, and 326 bacterial genera were identified, averagely 73.5 genera per sample.

### 2.2. Microbial Compositions in Bladder Cancer Samples

**Figure 1:**
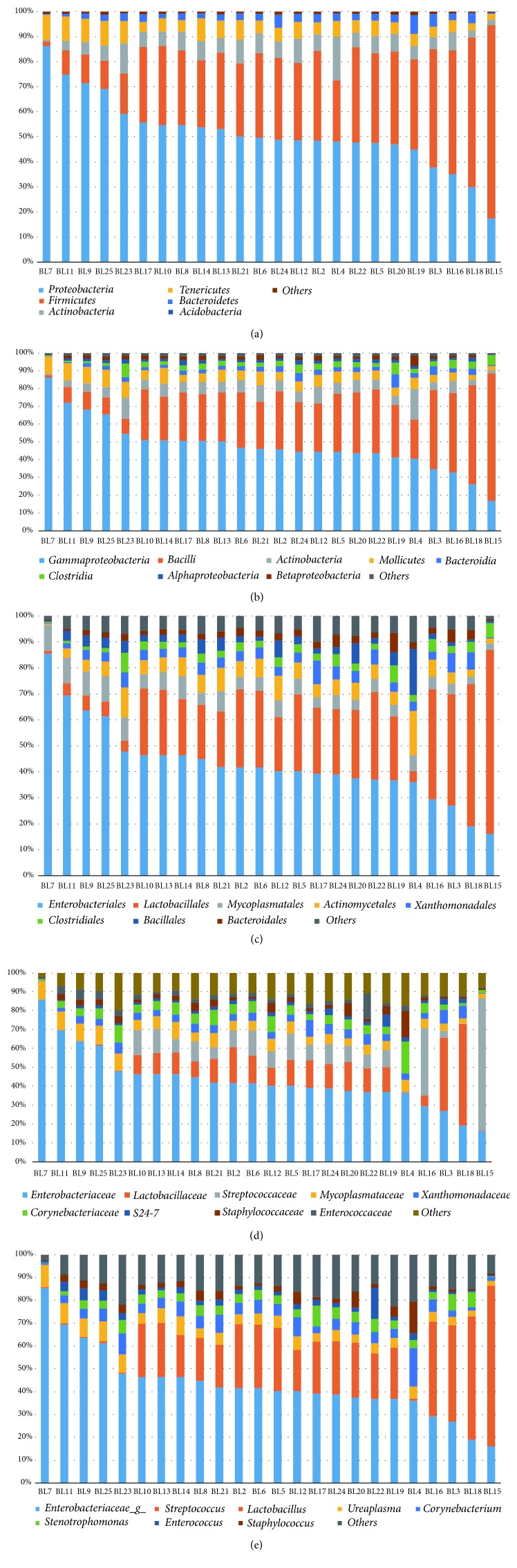
The urinary microbiota of bladder cancer patients. Most abundant taxa are shown at (a) phylum, (b) class, (c) order, (d) family, and (e) genus level.

We analyzed the microbial composition in bladder cancer at the levels of phylum, class, order, family, and genus. The five most abundant phyla are* Proteobacteria*,* Firmicutes*,* Actinobacteria*,* Tenericutes*, and* Bacteroidetes *(Figure 1(a)). In the study by Wu et al., the top phyla in the bladder cancer samples were* Proteobacteria*,* Firmicutes*,* Actinobacteria*, and* Bacteroidetes* [2]; and in the study by Bucevic et al., the top phyla in the bladder cancer samples were* Firmicutes*,* Actinobacteria*,* Bacteroidetes*, and* Proteobacteria* [1]. The phyla* Proteobacteria*,* Firmicutes*,* Actinobacteria*, and* Bacteroidetes* were abundant in bladder cancer samples from all three laboratories. The most abundant classes in the bladder cancer samples in our laboratory are* Gammaproteobacteria*,* Bacilli*,* Actinobacteria*,* Mollicutes*,* Bacteroidia*,* Betaproteobacteria*, and* Clostridia* (Figure 1(b)); in the study by Wu et al., the most abundant classes in the bladder cancer samples were* Gammaproteobacteria*,* Bacilli*,* Actinobacteria*, and* Betaproteobacteria* [2]; and in the study by Bucevic et al., the most abundant classes in the bladder cancer samples were* Clostridia*,* Actinobacteria*,* Bacteroidia*,* Bacilli*,* Epsilonproteobacteria*,* Gammaproteobacteria*,* Fusobacteria*, and* Synergistia* [1]. The classes* Gammaproteobacteria*,* Bacilli*, and* Actinobacteria* were abundant in bladder cancer samples from all three studies. The most abundant orders in the bladder cancer samples in our laboratory are* Enterobacteriales*,* Lactobacillales*,* Mycoplasmatales*,* Actinomycetales*,* Xanthomonadales*,* Clostridiales*,* Bacillales*, and* Bacteroidales* (Figure 1(c)); in the study by Wu et al., the most abundant orders in the bladder cancer samples were* Enterobacteriales*,* Bacillales*,* Lactobacillales*,* Corynebacteriales*, and* Bacteroidales* [2]; in the study by Bucevic et al., the most abundant orders in the bladder cancer samples were* Clostridiales*,* Actinomycetales*,* Bacteroidales*,* Lactobacillales*,* Campylobacterales*,* Enterobacteriales*,* Bacillales*, and* Fusobacteriales *[1]. The orders* Enterobacteriales*,* Bacillales*,* Lactobacillales*, and* Bacteroidales* were abundant in bladder cancer samples from all three laboratories. The most abundant families are* Enterobacteriaceae*,* Lactobacillaceae*,* Streptococcaceae*,* Mycoplasmataceae*,* Xanthomonadaceae*, and* Corynebacteriaceae* (Figure 1(d)). The most abundant genera are* Enterobacteriaceae_g_*,* Streptococcus*,* Lactobacillus*,* Ureaplasma*,* Corynebacterium*,* Stenotrophomonas*,* Enterococcus*, and* Staphylococcus *(Figure 1(e)).

### 2.3. The Core Bacteria in Bladder Cancer

**Figure 2:**
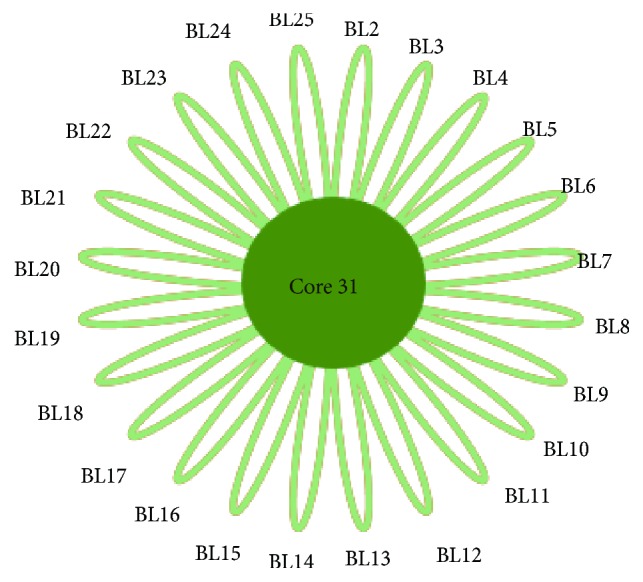
Common core bacteria in 24 bladder cancer samples. There are 31 bacterial genera (Core31) in all of these 24 samples, including* Clostridiales_f_g*,* Peptoniphilus*,* Mycoplasma*,* Cupriavidus*,* Lachnospiraceae*,* Ureaplasma*,* Delftia*,* o_Rhizobiales_f_g*,* Acinetobacter*,* Enterococcus*,* Hydrogenophilus*,* Prevotella*,* Bacillus*,* Brevundimonas*,* f_Enterobacteriaceae_g*,* Geobacillus*,* Streptococcus*,* f_S247_g*,* Rubrobacter*,* Bifidobacterium*,* Finegoldia*,* Achromobacter*,* Stenotrophomonas*,* Actinomyces*,* Lactobacillus*,* f_Oxalobacteraceae_g*,* Sphingomonas*,* Anaerococcus*,* Staphylococcus*,* Corynebacterium*, and* Sphingobacterium*. BLnum: the number of bladder cancer samples.

**Figure 3:**
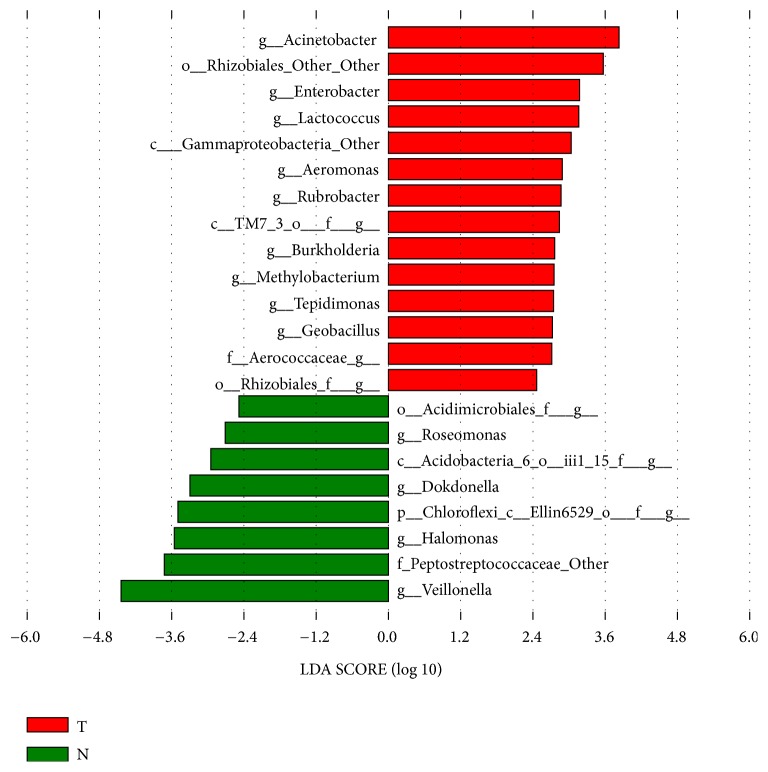
Microbial taxa associated with bladder cancer. The association of microbiota taxa with bladder cancer patients and healthy controls from other laboratories was analyzed by LEfSe. Red indicates taxa enriched in bladder cancer patients, and green indicates taxa enriched in healthy controls.

The common bacteria of the 24 bladder cancer samples were analyzed using the online website http://bioinformatics.psb.ugent.be/webtools/Venn/. We found 31 common core bacterial genera in these samples (Figure 2), that is,* Clostridiales_f_g*,* Peptoniphilus*,* Mycoplasma*,* Cupriavidus*,* Lachnospiraceae*,* Ureaplasma*,* Delftia*,* o_Rhizobiales_f_g*,* Acinetobacter*,* Enterococcus*,* Hydrogenophilus*,* Prevotella*,* Bacillus*,* Brevundimonas*,* f_Enterobacteriaceae_g*,* Geobacillus*,* Streptococcus*,* f_S247_g*,* Rubrobacter*,* Bifidobacterium*,* Finegoldia*,* Achromobacter*,* Stenotrophomonas*,* Actinomyces*,* Lactobacillus*,* f_Oxalobacteraceae_g*,* Sphingomonas*,* Anaerococcus*,* Staphylococcus*,* Corynebacterium*, and* Sphingobacterium*. Some of these common core bacteria may be inherently present in urine, and some may be associated with bladder cancer. In the study by Wu et al., the most abundant genera in the bladder cancer samples were* Escherichia*,* Shigella*,* Staphylococcus*,* Streptococcus*,* Aeromonas*,* Acinetobacter*,* Bacteroides*,* Lactobacillus*,* Serratia*,* Proteus*,* Laceyella*, and* Fusobacterium* [2]. The common core genera* Staphylococcus*,* Streptococcus*,* Acinetobacter*, and* Lactobacillus* were also abundant in bladder cancer samples from the study by Wu et al. [2]. Moreover, the common core bacteria* Acinetobacter*,* Rubrobacter*,* Geobacillus*, and* Rhizobiales* (Figure 3) are significantly more abundant in bladder cancer than in control samples (based on two previous datasets [1, 2]). These may include bacteria associated with bladder cancer, but this hypothesis requires further experimental verification.

### 2.4. Different Abundance of Core Bacteria between Bladder Cancer and Control Samples from Other Laboratories

Linear discriminant analysis effect size (LEfSe) was used to analyze bladder cancer and healthy control samples from other laboratories [2, 13]. Fourteen bacterial genera were found to be significantly higher abundant in bladder cancer samples than in the control group (Figure 3). Among these, four genera including* Acinetobacter*,* Rubrobacter*,* Geobacillus*, and* Rhizobiales* were also found in the core bacteria of our 24 bladder cancer samples.

**Figure 4:**
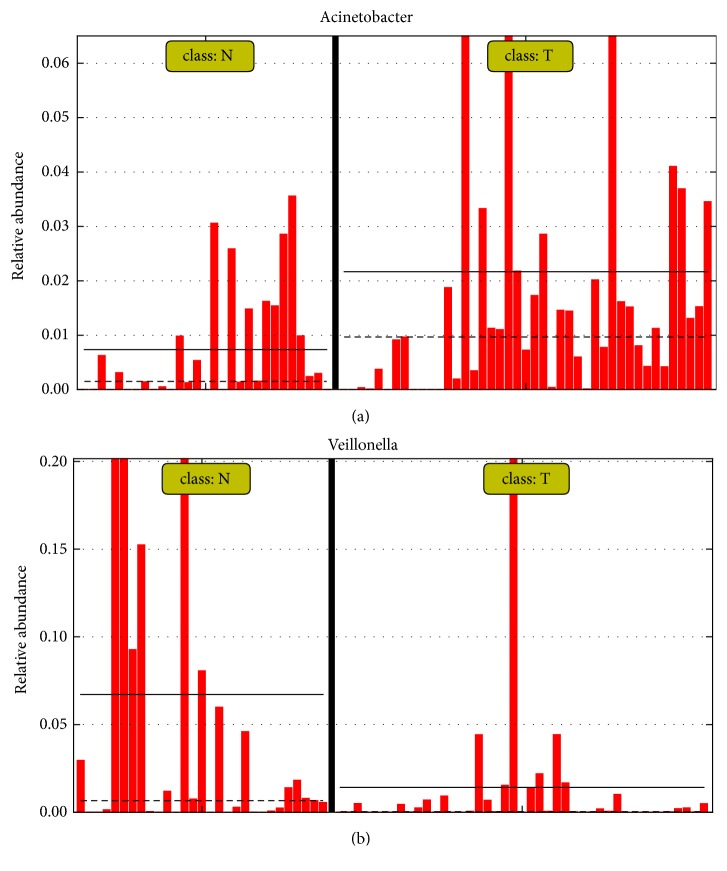
Different relative abundance of (a)* Acinetobacter *and (b)* Veillonella* in bladder cancer and healthy control samples.

In particular,* Acinetobacter* abundance was significantly higher in bladder cancer samples than in the control group (based on two previous datasets [1, 2]) (Figures 3 and 4(a)). Interestingly,* Acinetobacter* was also reported to be significantly more abundant (*P* = 0.048) in bladder cancer patients in the study by Wu et al. [2]. It was reported that* Acinetobacter *spp. are among the most abundant Gram-negative bacteria isolated from bovine urine affected by bladder urothelial tumors [14]. In addition, it was reported that the prevalence of* Acinetobacter baumannii *in gastric cancer patients was significantly higher than in nonulcer dyspepsia and peptic ulcer patients (25% vs. 3.2% and 4.5%) [15].* Acinetobacter* is a complex genus, which is associated with nosocomial infections, including urinary tract infections [2].* A. baumannii* has been used as model biofilm forming bacteria [16]. Biofilms, in which the microbial communities are embedded in a biopolymer matrix, are especially interesting, because they are the main cause of human bacterial infections and have a strong resistance to antibiotics and host immunity [17–21]. Recent studies have found that bacterial biofilms play a role in the onset and development of various cancers, including stomach cancer [22], colorectal cancer [23], colon cancer [24], prostate cancer [25], and lung cancer [26].* A. baumannii* is not only involved in the formation of biofilm but also involved in the adhesion and invasion of epithelial cells, the spread of bacteria caused by the degradation of phospholipids in the mucosal barrier, and the escape of the host immune response [27]. Therefore,* Acinetobacter* may be related to bladder cancer and is a potential microbial marker of bladder cancer, but a potential causal relationship between* Acinetobacter* abundance and bladder cancer requires further experimental verification. Interestingly,* Veillonella* abundance was significantly lower in bladder cancer samples than in the control group (based on two previous datasets [1, 2]); this needs to be further explored in the future (Figures 3 and 4(b)).

## 3. Conclusion

In this study, we characterized the urinary microbial profile of bladder cancer by 16S rRNA gene sequencing. The results reveal 31 common core bacteria from 24 bladder cancer samples collected in our laboratory. In addition, four common core bacteria are significantly more abundant in bladder cancer samples than in nondiseased people (based on two previous datasets [1, 2]). Our study suggests that some common core bacteria, especially* Acinetobacter*, may be associated with bladder cancer, but the causal relationship is not yet clear. A better understanding of the role of urinary microflora in the onset and development of bladder cancer can provide novel biomarkers for diagnosis and prognosis, as well as more microbial-targeted therapeutic strategies. There are still some limitations of our research. Firstly, a causal relationship between microorganisms and bladder cancer has not been elucidated, and the number of samples is small. Therefore, a larger number of prospective follow-up studies and animal experiments are needed to clarify the role of microorganisms in the onset and development of bladder cancer. Another limitation is that although the 16S rRNA gene sequencing method enables us to detect bacteria that exist in low numbers it cannot detect bacteria at the species level or nonbacterial microorganisms, such as viruses and fungi [5]. The future need to sample at other regions in order to understand the skewed results [28] from different regions by comparing cases and controls. The same problem can happen if samples were processed with different experimental procedures (sample preparation, DNA extraction, PCR, and etc.). The future need to sample at other regions in order to understand the skewed results [28] from different regions by comparing cases and controls.

## 4. Materials and Methods

### 4.1. Subject Recruitment and Sample Collection

The research began with the approval of the Ethics Committee of Shenzhen Institutes of Advanced Technology, Chinese Academy of Sciences. Urine samples were collected from 25 patients with bladder cancer, 18 males and 7 females, in the First Affiliated Hospital of Zhengzhou University, between January 2017 and March 2017. After the initial suspicion of urinary bladder cancer, the urologist carried out a physical and an ultrasound examination. Then the cancer tissue was removed by the transurethral resection (TUR) approach, and the diagnosis was confirmed by the pathologist after cystoscopy and tumor tissue examination. Urine samples were collected after ultrasound examination and before cystoscopy. The characteristics of bladder cancer patients are given in Table S1. All of the experiments were conducted in accordance with relevant guidelines and regulations, and participants gave written informed consent for urine collection and analysis for research purposes. Clean catch, midstream urine was collected from the bladder cancer patients and stored at −80°C until DNA extraction.

### 4.2. DNA Isolation from Urine

The genomic DNA of urinary bacteria was extracted using an EasyPure Bacteria Genomic DNA Kit (TRANSGEN). The procedure is as follows: 20 ml urine sample was collected in a 50-ml sterile centrifugal tube and centrifuged at 10,000 rpm for 15 minutes. The supernatant was discarded. The pellet was transferred to a centrifugal tube of 1.5 ml and centrifuged at 13,500 rpm for 10 minutes. The supernatant was discarded. Lysis buffer containing lysozyme was added to the centrifugal tube. The sample was incubated at 37°C for 1 hour and centrifuged at 13,500 rpm for 1 minute, and the supernatant was discarded. The protein fraction was digested by adding protease K at 55°C for 15 minutes. RNaseA was added to digest RNA. Absolute ethanol was added to dehydrate and precipitate the DNA. The solution was added to a centrifugal column and centrifuged at 13,500 rpm for 30 seconds. The effluent was discarded. Washing buffer was used to clean the DNA suspended in the centrifugal column. Finally, DNA was eluted with deionized water. The concentration of DNA was measured with Nanodrop.

### 4.3. 16S rRNA Gene Amplification and Hi-Seq Sequencing

The V4 region of the 16S rRNA gene was amplified by 515F-806R fusion primers that included a linker and indexing barcodes. The F515 primer (5′TATGGTAATTGTGTGCCAGCMGCCGCGGTAA3′) was used for all of the samples. We added the same linker and different barcode sequences at the 5′ end of the R806 primer (5′AGTCAGTCAGCCGGACTACHVGGGTWTCTAAT3′) (Table S1). PCRs were run in a final volume of 50 *μ*l, containing 2 *μ*l of DNA as template, 2 *μ*l of F515 primer, 2 *μ*l of R806 primer, 4 *μ*l of dNTPs, 4 *μ*l of 25 mmol l-1 MgCl_2_, 5 *μ*l of 10× Ex Taq buffer, 0.25 *μ*l Taq polymerase (5 U/*μ*l), and 30.75 *μ*l of distilled deionized water. PCR started at 98°C for 1 min, followed by 30 cycles of 98°C for 10 s, 58°C for 30 s, and 72°C for 2 min and one final elongation step at 72°C for 10 min. The PCR products were purified by the SanPrep Column DNA Gel Extraction Kit (Sangon Biotech, Shanghai, China) and preserved in 25 *μ*l sterile water. The concentrations of purified PCR products were determined by NanoDrop (Thermo Fisher Scientific Inc., Waltham, MA, USA); 200 ng of the purified PCR products of each sample was added together in equal amounts and then sent to Illumina Hi-Seq2500 V4 platform of Novogene Genomics Co., Ltd. (Beijing) for sequencing.

### 4.4. Bioinformatics and Statistical Analyses

Raw data, including bladder cancer and healthy control samples from other laboratories [2, 13], were filtered to eliminate adapter contamination and low quality reads by using QIIME [12]. The filtered sequences were clustered into the OTU with 97% identity, using QIIME [12], and then a representative sequence from each clustered OTU was used to align to the Greengenes Database [29]. QIIME was used to evaluate alpha diversity, which was based on the observed species and the Shannon index. To identify significant differences in bacterial abundance in bladder cancer and healthy control samples from other laboratories [2, 13], taxon summaries at the genus level were reformatted and analyzed by LEfSe [30]. R software was used to find the intersection of the core bacteria of these 24 bladder cancer samples and significantly different bacteria between bladder cancer and healthy control samples.

## Data Availability

The datasets generated during the current study are available from the NCBI SRA database with accession numbers SRR8647671.

## Supplementary Materials

Supplementary Materials Figure S1: rarefaction curves. It can be seen that the Shannon index of the 24 samples has reached saturation, indicating that the sequencing depth is sufficient, and most samples have captured complete microbial profiles. Table S1: characteristics of 24 patients with bladder cancer.
